# Synergistic targeting of malignant pleural mesothelioma cells by MDM2 inhibitors and TRAIL agonists

**DOI:** 10.18632/oncotarget.17790

**Published:** 2017-05-11

**Authors:** Loredana Urso, Ilaria Cavallari, Micol Silic-Benussi, Lorena Biasini, Giulia Zago, Fiorella Calabrese, Pier Franco Conte, Vincenzo Ciminale, Giulia Pasello

**Affiliations:** ^1^ Department of Surgery, Oncology and Gastroenterology, University of Padova, 35128, Padova, Italy; ^2^ Immunology and Molecular Oncology Unit, Veneto Institute of Oncology, IRCCS, 35128, Padova, Italy; ^3^ Medical Oncology Unit 2, Veneto Institute of Oncology, IRCCS, 35128, Padova, Italy; ^4^ Department of Cardio-Thoracic and Vascular Sciences, University of Padova, 35128, Padova, Italy

**Keywords:** mesothelioma, MDM2, p53, RG7112, TRAIL

## Abstract

Malignant Pleural Mesothelioma (MPM) is a chemoresistant tumor characterized by low rate of p53 mutation and upregulation of Murine Double Minute 2 (MDM2), suggesting that it may be effectively targeted using MDM2 inhibitors. In the present study, we investigated the anticancer activity of the MDM2 inhibitors Nutlin 3a (*in vitro*) and RG7112 (*in vivo*), as single agents or in combination with rhTRAIL.

*In vitro* studies were performed using MPM cell lines derived from epithelioid (ZL55, M14K), biphasic (MSTO211H) and sarcomatoid (ZL34) MPMs. *In vivo* studies were conducted on a sarcomatoid MPM mouse model.

In all the cell lines tested (with the exception of ZL55, which carries a biallelic loss-of-function mutation of p53), Nutlin 3a enhanced p21, MDM2 and DR5 expression, and decreased survivin expression. These changes were associated to cell cycle arrest but not to a significant induction of apoptosis. A synergistic pro-apoptotic effect was obtained through the association of rhTRAIL in all the cell lines harboring functional p53. This synergistic interaction of MDM2 inhibitor and TRAIL agonist was confirmed using a mouse preclinical model. Our results suggest that the combined targeting of MDM2 and TRAIL might provide a novel therapeutic option for treatment of MPM patients, particularly in the case of sarcomatoid MPM with MDM2 overexpression and functional inactivation of wild-type p53.

## INTRODUCTION

Malignant Pleural Mesothelioma (MPM) is a rare cancer with poor prognosis and increasing incidence [1]. The sarcomatoid (16%) and biphasic (34%) histologic subtypes are characterized by a worse prognosis compared to epithelioid MPM (50%) [2].

Surgery is feasible in few selected patients, and current systemic chemotherapy does not yield satisfactory results. Standard chemotherapy with a platinum-based doublet *plus* an antifolate drug achieves median overall survival (OS) and progression free survival (PFS) of about 12 and 6 months, respectively [3, 4]. The lack of effective second line treatments and the failure of targeted therapies reinforce the need for new molecular targets and drugs for MPM treatment.

The molecular pathogenesis of MPM is characterized by frequent deletion of the INK4A/ARF locus (70–80%), which encodes p14/ARF and p16/INK4A, while p53 is not mutated in the majority of the cases [5, 6]. p14/ARF, an inhibitor of Murine Double Minute 2 (MDM2), is crucial in the control of cell proliferation [7]. In unstressed cells MDM2 and MDMX (also known as MDM4) keep p53 expression at very low levels through different mechanisms which include ubiquitylation and proteasomal degradation [8]. MDM2 expression is, in turn, enhanced by p53, thus creating a powerful negative feedback loop [9]. In the absence of genetic alteration, p53 function may be lost as a consequence of MDM2 or MDMX overexpression, an alteration that is present in several solid and hematological tumors and correlates with poor prognosis and resistance to therapy [10, 11]. MDM2 overexpression and p53 mutations thus seem to be mutually exclusive, while tumors overexpressing MDMX may also carry inactivated p53 [12]. In addition, MDM2 and MDMX may affect proliferation, angiogenesis, and DNA repair through p53-independent mechanisms [7, 12, 13]. To date, several MDM2 inhibitors have been tested in preclinical studies and in clinical trials. Among these, the most studied are Nutlin 3a and its analog, specifically designed for clinical use, RG7112 (RO5045337, Roche, Basel, Switzerland). p53 reactivation by MDM2 inhibitors sensitizes p53 wild-type cancer cells to DNA damaging agents, which trigger the intrinsic pathway of apoptosis, or to extrinsic apoptosis activators such as TRAIL (Tumor necrosis factor (TNF)-related apoptosis-inducing ligand) [14–17]. Nutlin 3a and RG7112 are particularly effective in cancer cells overexpressing MDM2, while they are ineffective in cancer cells with downstream defects in the p53 pathway [18] or in cells overexpressing MDMX [19]. MDM2 overexpression has been previously reported in MPM samples, especially in the sarcomatoid and biphasic subtypes [20, 21], where it might represent a promising therapeutic target.

Building on these findings, in the present study we investigated the combination of rhTRAIL and Nutlin 3a or RG7112, in p53 wild type MPM cells, *in vitro* and in a preclinical MPM model.

## RESULTS

### p53 status and MDM2 expression in MPM cell lines

Mutational analysis of p53 in the MPM cell lines revealed a missense mutation in exon 7 (c.725G>A) associated with loss of heterozygosity (LOH) in ZL55 cells, M14K cells carried a monoallelic frameshift mutation in exon 5 (c.406delC), while MSTO 211H and ZL34 were not mutated (Table 1). Based on the IARC and Cosmic database (http://www.iarc.fr; http://cancer.sanger.ac.uk/cosmic), the 725G>A mutation causes loss of function of p53 and the 406delC, by inducing a frameshift, results in a premature stop codon located in exon 5 and in the expression of a truncated protein. qRT-PCR and immunoblot analysis revealed that MDM2 mRNA and protein levels, were higher in the ZL34 cell line, which carries a wild-type p53 (Supplementary Figure 1A).

**Table 1:** p53 status in MPM cell lines

MPM Cell Lines	Histology	TP53 Status	Nucleotide change	AA Mutation	TA Class
**ZL55**	Epithelioid	Mutated	c. 725G>A (exon 7)	p.C242Y (substitution-Missense)	Non Functional
**M14K**	Epithelioid	Mutated (monoallelic)	c.406delC (Exon 5)	p.Q136fs*34(Deletion-Frameshift)	Unknown
**MSTO211H**	Biphasic	Wild Type	-	-	
**ZL34**	Sarcomatoid	Wild Type	-	-	

### Nutlin 3a activates p53 and induces cell cycle arrest in MPM cell lines

**Figure 1:**
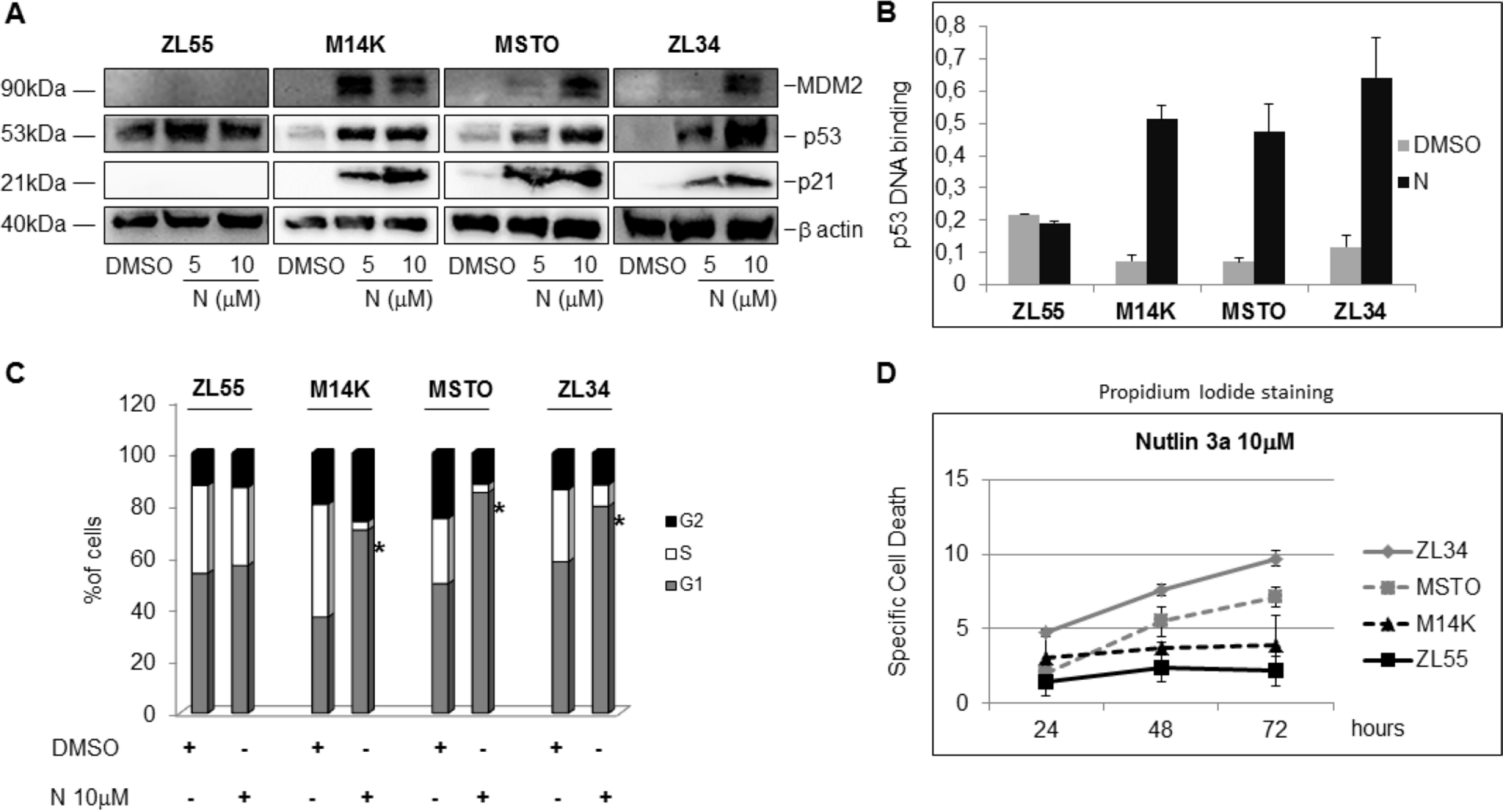
Nutlin 3a activity in p53 wild type and p53 mutated MPM cell lines (**A**) Western blot analysis to detect MDM2, p53, p21 and β actin in MPM cell lines treated with DMSO or Nutlin 3a (N) (5 and 10 μM) for 24 hours. The figure is representative of two independent experiments. (**B**) p53 binding assay carried out using nuclear extracts of MPM cell lines treated with DMSO or Nutlin 3a 10 μM for 24 hours. Bar graph represents mean ± SD of the OD450 nm adsorbance values of two independent experiments (each run in duplicate). (**C**) Cell Cycle analysis of MPM cell by DNA content measurement using propidium iodide staining and flow cytometry. Cells were treated with DMSO or Nutlin 3a 10 μM for 24 hours. The graph represents the mean of the percentage of cells in each phase of the cell cycle of three different experiments (each run in duplicate). The asterisk indicates statistically significant (*p* < 0.05) differences of the percentage of cells in G1 phase between mock-treated (DMSO) and Nutlin 3a-treated MPM cells. *p* values were calculated using the Mann-Whitney runk sum test. (**D**) Specific Cell Death of MPM cell lines treated with DMSO or Nutlin 3a 10 μM for 24, 48 and 72 hours. Results were represented as mean ± SD of two independent experiments, each run in duplicate. Specific Cell Death was calculated as detailed in Materials and Methods.

We next analyzed the effects of Nutlin 3a on the expression of MDM2 and p21, two p53 transcriptional targets. Although p53 accumulation occurred in all the MPM cell lines upon treatment with Nutlin 3a, a dose dependent induction of p21 and MDM2 was detected in M14K, MSTO211H and ZL34 cells (p53 WT), but not in ZL55 cells (p53 −/−) (Figure 1A). Furthermore, DNA binding assays showed a strong increase in p53 binding to its target DNA in M14K, MSTO211H and ZL34 (but not ZL55) cell lines (Figure 1B).

Consistent with a p53-dependent induction of p21, Nutlin 3a induced an accumulation in the G1 phase of the cell cycle in M14K, MSTO211H and ZL34 cells, but not in ZL55 (Figure 1C).

### Nutlin 3a synergizes with rhTRAIL in MPM cell lines

**Figure 2:**
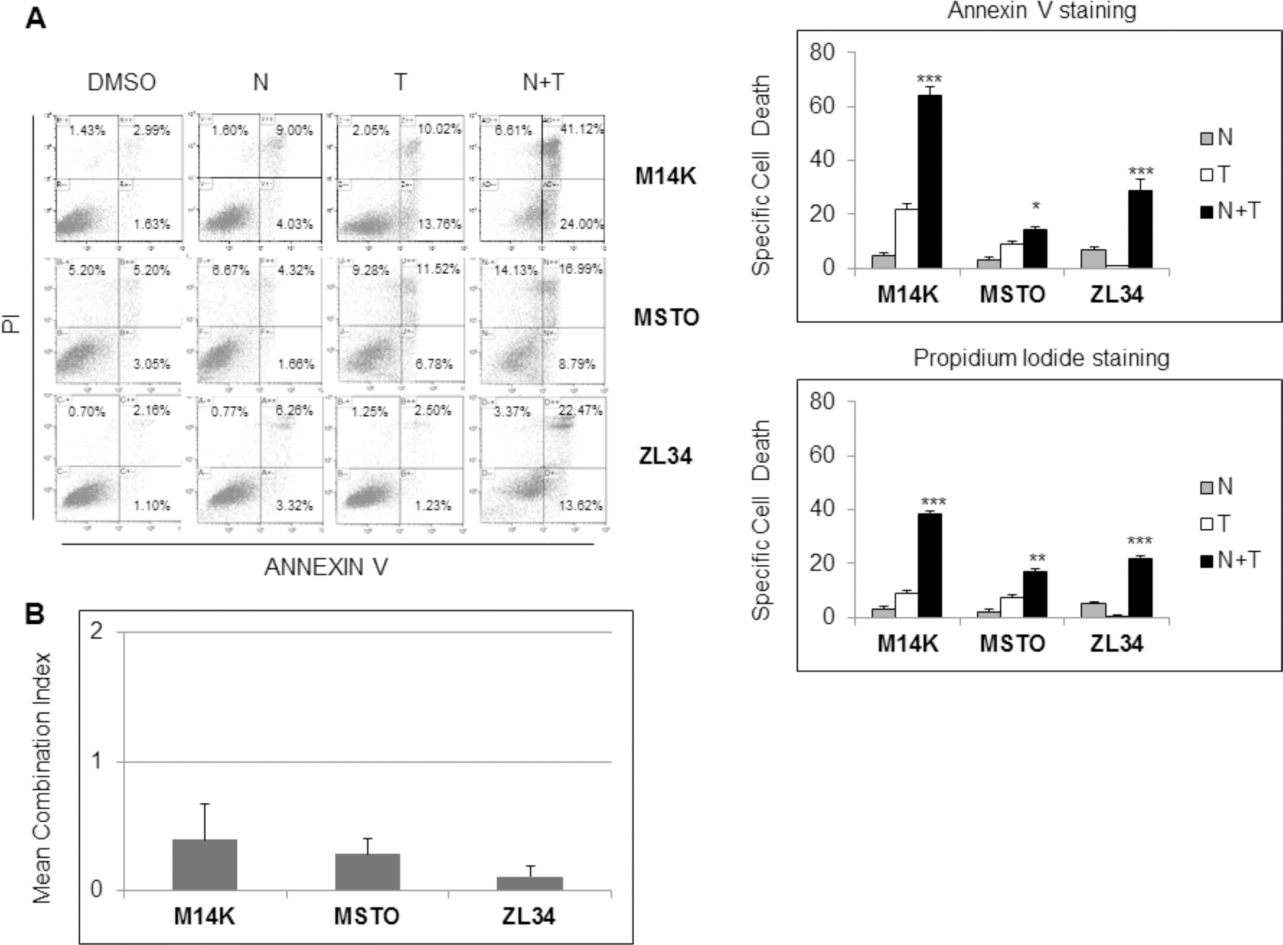
Nutlin 3a synergizes with rh/TRAIL in apoptosis induction in MPM cell lines (**A**) Biparametric Annexin V/propidium iodide flow cytometry analysis to test apoptosis of MPM cell lines treated with 10 μM Nutlin 3a and/or 2.56 μM rhTRAIL (T) for 24 hours. A representative dot blot is shown (left panel). Results are represented as mean ± SE of three independent experiments run in triplicate (right panel). Asterisks indicate statistically significant differences between MPM cells treated with rhTRAIL compared with cells treated with Nutlin 3a *plus* rhTRAIL (*p < 0.05; **p < 0.005 and ***p < 0.001); *p* values were calculated using the Mann-Whitney runk sum test. (**B**) Isobologram analysis using the Chou-Talalay equation. MPM cell lines were treated for 24 hours with Nutlin 3a and rhTRAIL (3.9:1 ratio). The synergistic effects of these drugs were assessed by calculating the Combination Index (CI) method (see Materials and Methods). Bar graph represents the mean ± SD of CI values at FA (fraction affected) 0.25, 0.5, 0.75 and 0.9.

Although inducing cell cycle arrest, Nutlin 3a induced only a modest increase in cell death of the MPM cell lines (Figure 1D). Since it was previously reported that p53 activation increases TRAIL induced apoptosis [15], we tested the effects of Nutlin 3a *plus* rhTRAIL in the p53 wild-type MPM cell lines. As expected, the combined treatment of Nutlin 3a and rhTRAIL resulted in a significant increase of Specific Cell Death (both Annexin V/PI double positive cells) (Figure 2A). Moreover, time course experiments showed a strong increase of the Specific Cell Death over time in ZL34 cells, but not in ZL55 cells (Supplementary Figure 2).

Isobologram analysis using the Chou and Talalay equation [22] revealed that the association of Nutlin 3a and rhTRAIL was strongly synergistic in M14K, MSTO 211H and ZL34 cells with a combination index (CI) < 1 (Figure 2B and Supplementary Figure 1B).

**Figure 3:**
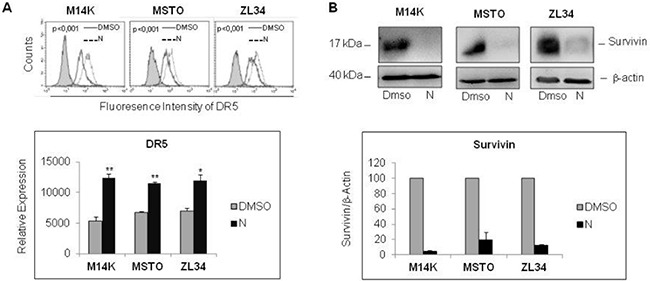
Nutlin 3a treatment increases DR5 and decreases Survivin expression (**A**) Flow cytometry analysis of DR5 expression in MPM cell lines treated with Nutlin 3a 10 μM for 24 hours. Representative histogram plots are shown (upper panel). *P* values were calculated using the Kolmogorov-Smirnov Statistics. The bar graph (lower panel) represents the mean ± SE of relative expression (calculated as detailed in Materials and Methods) of DR5 of three independent experiments run in triplicate. Statistically significant differences of receptor expression between untreated (DMSO) MPM cells compared to MPM cells treated with Nutlin 3a were evaluated using the Mann-Whitney runk sum test (*p < 0.05; **p < 0.005). (**B**) Western Blot analysis for survivin expression of MPM cell lines treated with Nutlin 3a 10 μM for 24 hours. A representative immunoblot is shown (upper panel). Densitometry of survivin values were normalized on β-actin values and reported as percentage relative to control (DMSO-treated samples). Mean values ±SE of three independent experiments are shown (lower panel).

**Figure 4:**
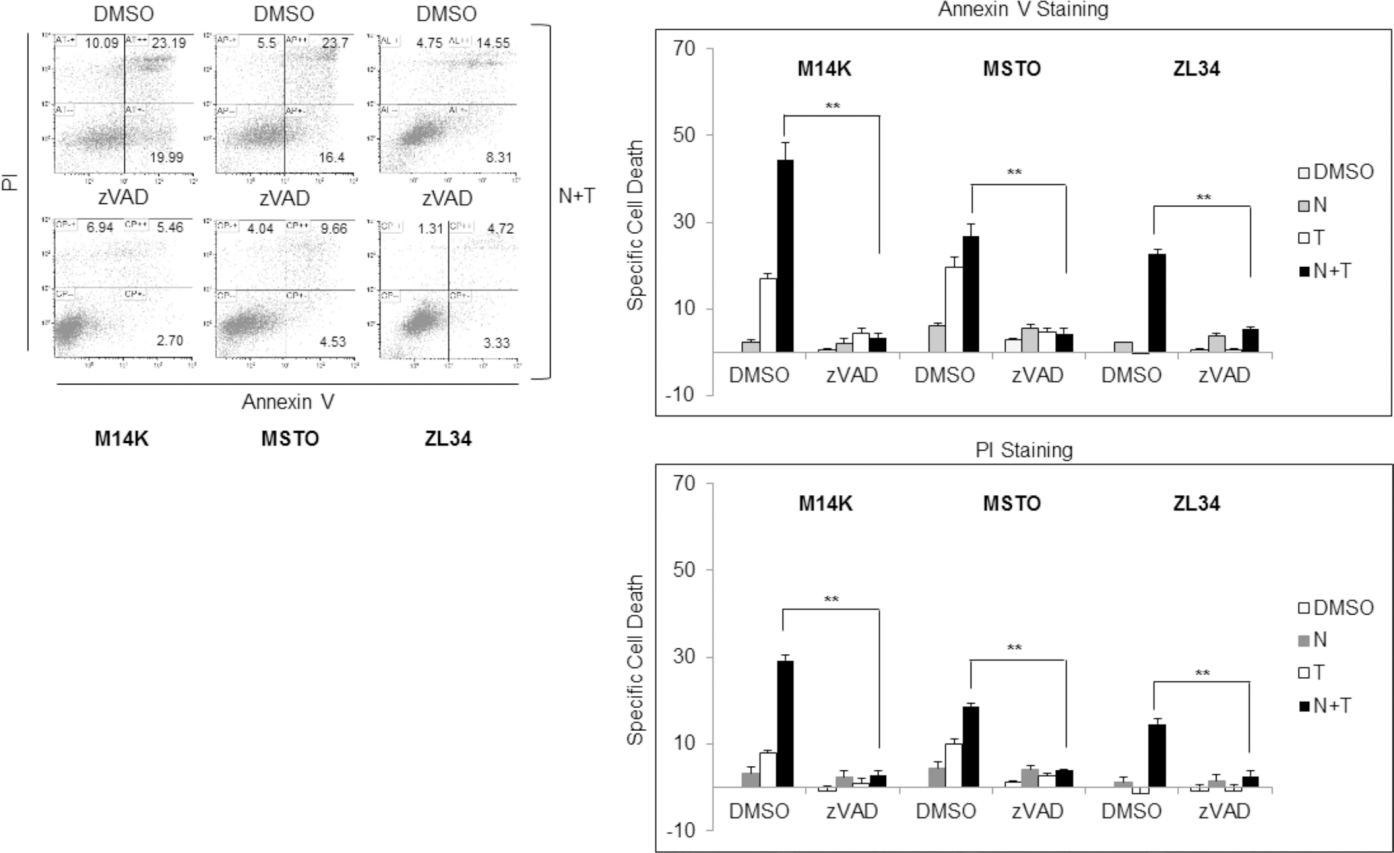
Nutlin 3a synergism with rh/TRAIL involves increase of apoptosis Apoptosis Assay of MPM cell lines treated with Nutlin 3a 10 μM and/or rhTRAIL (T) 2.56 μM for 24 hours, in presence or absence of 1 hour zVAD-fmk preincubation. Results are represented as mean ± SE of two independent experiments, each run in triplicate (right panel). Asterisks indicate statistically significant difference between Nutlin 3a *plus* rhTRAIL-treated cells in absence or presence of zVAD (*p* < 0.005) calculated using the Mann-Whitney runk sum test. In the left panel a representative dot blot is shown.

The synergistic interaction of Nutlin 3a and TRAIL is likely to result from the bidirectional connections between TRAIL and p53. TRAIL can engage the mitochondrial/intrinsic apoptotic pathway through caspase-8-mediated cleavage of Bid [23], and p53 may enhance responsivity to TRAIL by increasing DR5 transcription [16, 17, 24–26] and decreasing survivin expression [15]. Consistent with these findings, our results showed that Nutlin 3a increased DR5 and decreased survivin expression in M14K, MSTO211H and ZL34 cells (Figure 3A, 3B). Consistent with this notion, when these MPM cells harbouring functional p53 were treated with Nutlin 3a and rhTRAIL, we observed a strong induction of cell death that was abrogated by treatment with the pan-caspase inhibitor zVAD-fmk (Figure 4), indicating that the apoptotic pathway was engaged.

### Antitumor activity of RG7112 plus rhTRAIL *in vivo*

The anticancer activity of MDM2 inhibition *plus* rhTRAIL treatment was tested *in vivo* by inoculating ZL34 cells intraperitoneally (IP) into SCID male mice. As Nutlin 3a is not suitable for clinical use because of its pharmacokinetics properties, we used its analogue RG7112, which is specifically formulated for clinical use. RG7112 efficiently inhibited MDM2-p53 binding and showed good pharmacological features [18].

**Figure 5:**
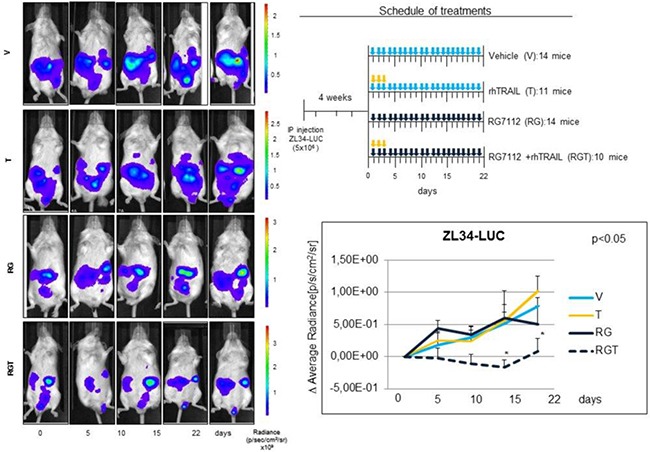
*In vivo* antitumor activity of RG7112 plus rhTRAIL in MPM xenograft mouse model *In vivo* experiments were conducted using SCID mice injected IP with 5 × 10^6^ ZL34-LUC. 4 weeks post injection mice were randomized in 4 treatment groups and treated with RG7112 (RG) or vehicle (V) (days 1–21) by gavage and/or rhTRAIL (T) (days 1–3) IP (see schedule of treatment). Tumor size was assessed at the indicated time point by *in vivo* bioluminescence. Left panel shows luminescence of a representative mouse for each group. The mean ± SE of the Δ Average radiance for each group at every time point of three independent experiments is shown in the graph. Asterisks indicate statistically significant differences of average radiance between treated groups and untreated controls (*p* < 0.05) using the one way ANOVA on ranks analysis followed by Dunn's post hoc test.

ZL34 were selected as a preclinical model on the basis of their higher MDM2 expression and stronger synergistic response to the Nutlin 3a and rhTRAIL combination *in vitro*. The cells were engineered with a lentiviral vector encoding the luciferase gene (ZL34-LUC) to allow non invasive tracking of tumor growth *in vivo*. RG7112 and rhTRAIL were administered as depicted in the treatment schedule shown in Figure 5.

Results showed a significant (*p* < 0,05) reduction of tumor growth in the RGT (RG7112 *plus* rhTRAIL) groups compared to mock-treated at the 15th and 22th days time points, while we observed no antitumor effect of RG7112 and TRAIL administered as single agents.

## DISCUSSION

Malignant Pleural Mesothelioma is a highly lethal disease, which is poorly responsive to current therapies [3, 4], particularly for the sarcomatoid subtype [27].

Clinical trials of biologic agents targeting key oncogenic pathways, such as phosphatidylinositol3-kinase (PI3K)/mammalian target of Rapamycin (mTOR), histone deacetylases (HDAC), Nuclear Factor kB (NFkB) and neoangiogenesis, did not achieve encouraging results and the systemic chemotherapy of MPM did not improve in the last 15 years. The lack of reliable biomarkers useful to select the patients more likely to benefit from specific treatments might, at least in part, explain these failures [28]. New therapeutic options and effective biomarkers for patient stratification/selection are thus in high demand.

In recent years, small molecule inhibitors of MDM2 or MDM2-p53 interaction have been investigated in several studies. Among these, the most studied are Nutlin 3a and RG7112. Compared to Nutlin 3a, RG7112 is more suited for *in vivo* use as it is less sensitive to oxidation, has lower molecular weight, shows stronger MDM2 binding affinity and more favourable pharmacokinetic properties [18]. Clinical trials revealed a modest effectiveness of RG7112 in solid tumors, most of which carry wild-type p53 [29]. However, RG7112 showed promising activity in leukemias [30] and a more potent nutlin analog, RG7388 (idasanutlin) [31, 32], is under investigation for the treatment of relapsed/refractory AML in a phase 3 trial (www.clinicaltrials.gov). A phase 1b clinical trial in sarcomas showed that the combination of RG7112 with doxorubicin produced significant haematological toxicity (increased neutropenia and thrombocytopenia) (Chawla SP et al., J Clin Oncol 31, 2013-abstr 10514). Drugs with a milder toxicity profile, such as rhTRAIL (Dulanermin) may thus be better suited for the association with MDM2 inhibitors [33].

rhTRAIL is a member of the TNF ligand superfamily, able to selectively kill transformed cells. TRAIL acts by binding the TRAIL Death Receptors 1 and 2 (DR4 and DR5) and activating both the extrinsic and the intrinsic apoptotic pathway. Although, several cancer cells show resistance to TRAIL [23], previous studies showed that DNA damaging agents or Nutlin 3a increased TRAIL mediated cell death through the increase of the TRAIL receptors expression [24–26, 34, 35]. To date, no data are available about the *in vivo* efficacy of this combination.

We reasoned that MPM might be a good model to test this combination because of its frequent MDM2 overexpression [20] and low rate of p53 mutation [36]. Importantly, higher expression levels of MDM2 characterize sarcomatoid/biphasic compared to epithelioid MPM samples [21].

Consistent with previous data [37], in our study no p53 activity was detected in p53 mutated ZL55 upon Nutlin 3a administration, while it was functional both in p53 WT cells (ZL34 and MSTO), and in M14K cell line harboring a monoallelic 406delC, thus suggesting that the other allele was sufficient to sustain p53 function (Figure 1A, 1B).

Nutlin 3a caused time-dependent induction of cell death in ZL34 and MSTO211H, but only minor effects in ZL55 and M14K cell line up to 72 hours of treatment (Figure 1D), probably because of higher expression of MDM2 and functional p53 in the first two cell lines.

Interestingly, when Nutlin 3a and rhTRAIL were simultaneously administered in p53 wild type cells, a synergistic increase of cell death was detected after 24 hours treatment (Figure 2). In p53-responsive MPM cells, Nutlin 3a induced DR5 expression, repression of survivin and increased apoptosis (Figures 3 and 4). The *in vivo* relevance of the combined actions of MDM2 inhibition and TRAIL agonists was tested using a xenograft mouse model that recapitulates sarcomatoid MPM with WT p53. Interestingly, our results demonstrated that RG7112 *plus* rhTRAIL significantly reduced tumor growth compared to the mock-treated group (Figure 5).

Taken together our data suggest that MDM2 inhibition plus TRAIL agonists may prove to be effective for MPM treatment, particularly (but not only) for non epithelioid MPM. p53 wild-type status and high expression levels of MDM2 could be critical to achieve a good response rate and so may represent important markers for patients’ selection.

## MATERIALS AND METHODS

### Cell lines and reagents

We employed two MPM cell lines of epithelioid derivation (ZL55, M14K), one biphasic cell line (MSTO211H) and one sarcomatoid cell line (ZL34). ZL55 and ZL34 cell lines were kindly supplied by Dr, E. Felley-Bosco (University of Zurich, Switzerland); M14K and MSTO211H cell lines were kindly supplied by Prof. L. Willems (University of Liège, Belgium). All cell lines have been authenticated by STR profile. Analysis was performed by BMR Genomics S.R.L (Padova, Italy). Analysis of ZL34 cell was performed on November 2015. Analysis of ZL55, M14K and MSTO211H was performed on February 2016. ZL55, ZL34 and MSTO211H cell lines displayed 96%, 100% and 100% match with reference profiles (www.phe-culturecollections.org.uk/; http://www.attc.org/) (evaluated by International Cell Line Authentication Committee – ICLAC- match criteria) (Supplementary Table 1). No reference profile is available for M14K cells.

All MPM cell lines were maintained in Roswell Park Memorial Institute medium (RPMI) 1640 (Gibco-Thermo Fisher Scientific, Waltham, Massachusetts, U.S.) supplemented with 2 mM L-glutamine, 1 mM sodium pyruvate, 10% FBS and 1% (w/v) penicillin/streptomycin (Invitrogen- Thermo Fisher Scientific, Waltham, Massachusetts, U.S.). All cells were cultured at 37°C in a humidified atmosphere containing 5% CO_2_. Nutlin 3a, was purchased from Sigma- Aldrich (Sigma-Aldrich St. Louis, MO, U.S.). RG7112 was supplied from Roche (Roche, Basel, Switzerland). rhTRAIL was supplied from Amgen/Genentech (Amgen Inc, Thousand Oaks, CA, U.S.; Genentech Inc, South San Francisco, CA, U.S.). Caspase Inhibitor I, zVAD-fmk, was purchased from Merck Millipore (Darmstadt, Germany).

### p53 mutational analysis

In the COSMIC, Catalogue of Somatic Mutations in Cancer, nine mutations are reported for 147 analysed mesotheliomas, and exons 5, 7, 8, 10 were involved. The IARC, International Agency for Research on Cancer, dataset also reported 10 TP53 mutated cases in exon 5, 7 and 8. Therefore, TP53 mutations were investigated by analysing exons 4 to 10, where the majority of mutations are localized.

Briefly, DNA isolated from MPM cell lines was subjected to PCR using primer pairs specific for each exon by the IARC protocol. The amplified products were then sequenced by fluorescent capillary electrophoresis (ABI PRISM 310 genetic analyzer, Applied Biosystems-Thermo Fisher Scientific, Waltham, Massachusetts, U.S.) and sequences were compared with NCBI Reference Sequence NC_000017.10.

### Western blot

Cells were washed twice in ice-cold PBS (phosphate buffer saline), and lysed in Mammalian Cells Disruption Buffer Paris-Kit (Ambion- Thermo Fisher Scientific, Waltham, Massachusetts, U.S.) supplemented with Phosphatase Inhibitor Cocktail (Roche, Basel, Switzerland) and Complete Protease Inhibitor Cocktail (Roche, Basel, Switzerland). Protein concentration was determined by the Coomassie (Bradford) Protein Assay Kit (Thermo Fisher Scientific, Waltham, Massachusetts, U.S.) using bovine serum albumin as standard, and equal amounts of proteins were analyzed by SDS-PAGE (acrylamide/bis-acrylamide). Gels were electroblotted onto polyvinylidenedifluoride membranes (Amersham-GE HEALTHCARE Little Chalfont, Buckinghamshire, U.K.). In immunoblot analysis, membranes were blocked for 1 hour with 5% non-fat dry milk in Tris Buffered Saline (TBS) containing 0.1% Tween-20, and incubated at 4°C over night with primary antibody direct against p53 (cat n°sc6243), MDM2 (cat n°sc 965), (Santa Cruz Biotechnology, Dallas, Texas, U.S.), p21 (cat n° 2946) (Cell Signaling Technology, Boston, MA, USA), survivin (cat n° ab76424) (ABCAM Cambridge, UK) and anti-β-actin antibody (cat n° A5060) (Sigma-Aldrich St. Louis, MO, U.S.) used as loading control, followed by horseradish peroxidase-conjugated secondary antibodies (Pierce-Thermo Fisher Scientific, Waltham, Massachusetts, U.S.). Finally, the membranes were incubated with chemiluminescence reagents (Lite Ablot Turbo; Euroclone, Milano, Italy) and revealed using UVITEC system (Uvitec, Cambridge, U.K.). Quantification of the bands was performed using UVITEC Image Quantification Software (Uvitec, Cambridge U.K.).

### p53 DNA-binding activity

p53 DNA-binding activity was assayed using the TransAM p53 kit (Active Motif Inc, Carlsbad, CA, U.S.), according to the manufacturer's instructions. Nuclear Extracts of MPM cells treated or not with Nutlin 3a 10 μM for 24 hours were used for the assay. p53 DNA binding was measured by colorimetric assay, and absorbance was read at 450 nm using Victor Microplate Reader (PerkinElmer Inc, Massachusetts, U.S.). Absorbance at 450 nm was proportional to p53 binding at the consensus DNA sequence.

### Cell cycle analysis

Cell cycle analysis was performed by flow cytometry detection of DNA content using Propidium iodide (PI) staining. MPM cells were treated with Nutlin 3a 10 μM. After 24 hours the cells were trypsinized, washed with PBS and fixed for 1 hour at –20°C in a following solution: RPMI (500 ml), FCS 10% (500 ml) and ice cold ethanol 70% (3 ml). After two washes in PBS, the cells were incubated with PBS, PI (Sigma-Aldrich St. Louis, MO, U.S.) 100 mg/ml and RNase (Qiagen, Venlo, Netherlands) 0,4 mg/ml for 1 hour at 37°C. PI staining was detected by FACSCalibur apparatus (BD Biosciences San Jose, CA, U.S.) and DNA content quantified using Modfit LT software (Verity, Topsham, Maine U.S).

### Apoptosis assay and drugs combination assay

MPM cells were seeded into 12-well plates in 1.0 mL/well of complete RPMI 1640 and treated with Nutlin 3a (10 μM) for 24, 48 and 72 hours and/or rhTRAIL 2.56 μM for 24 hours. Apoptosis Assay was performed using Annexin-V-Fluos and PI staining (Roche, Basel, Switzerland) according to the manufacturers’ instructions. Cells were collected, centrifuged, and resuspended in 300 μL of Annexin-binding buffer, followed by incubation with 1 μL of Annexin V-Fluos and 1μL of PI for 10 minutes at room temperature. Cells positive for Annexin V/PI were detected by flow cytometry using a FACSCalibur apparatus and analyzed using CellQuest software (BD Biosciences San Jose, CA, U.S.). Specific Cell Death was calculated by the following formula: (percentage of Annexin V or PI positive cells in treated samples- percentage of AnnexinV or PI positive cells in untreated samples)/(100- percentage of Annexin V or PI positive cells in untreated samples)* 100.

Drug Combination studies were conducted using the Chou-Talalay method [22]. MPM cell lines were treated for 24 hours with different concentration of Nutlin 3a and rhTRAIL (3.9:1 ratio) and Specific Cell Death was measured by PI staining.

The combination index (CI) was calculated using the CompuSyn software (ComboSyn, Inc., Paramus, NJ), where CI < 1, CI=1, and CI > 1 indicated synergistic, additive, and antagonistic effects, respectively.

### DR5 expression levels quantification

Surface expression of DR5 TRAIL receptor was evaluated by indirect immunostaining using a specific DR5 primary antibody (cat n° AG-20B-0023) (Alexis Biochemicals, San Diego, CA, U.S.) followed by Alexa Fluor 488 Goat anti-mouse immunoglobulin G (IgG H+L) (Life Technologies-Thermo Fisher Scientific, Waltham, Massachusetts, U.S.). Flow cytometry analysis was performed using a FACSCalibur apparatus and CellQuest software (BD Biosciences San Jose, CA, U.S.). Relative expression of TRAIL-R was calculated by the following formula: percentage of positive cells x mean fluorescence intensity (MFI).

### *In vivo* experiments

*In vivo* experiments were performed in accordance with the Padua University Ethic Committee for Animal Testing. 49 SCID male mice at the 6^th^ week were intraperitoneally (IP) injected with 5 × 10^6^ ZL34 cells previously transduced with lentiviral vector containing a plasmid encoding for Luciferase (ZL34-LUC). 4 weeks post injection mice were randomized in 4 treatment groups and treated with RG7112 (RG, 100 mg/Kg/die on days 1–21) or vehicle (V, 100 ml on days 1–21) by gavage and/or rhTRAIL (T, 60 mg/Kg/die on days 1–3) IP. rhTRAIL and RG7112 schedule and dose were established according to previous studies (data on file, Amgen Inc, CA/Genentech Inc, 2009 and [18]). Tumor size was assessed by *in vivo* bioluminescence using Xenogen bioluminescence imaging (IVIS, Xenogen, Alameda, CA, U.S.) after IP injection of D-luciferin (150 mg/Kg) in each mouse at the indicated time point. Mice were suppressed at the 22th day. Average Radiance [p/s/cm^2^/sr] was proportional to the number of ZL34-LUC cells. Δ Average radiance was used as indicator of tumor growth and calculated by the following formula: (Average Radiance at the day n- Average Radiance at the day 1)/Average radiance at the day 1.

### Statistical analysis

All data were analyzed using the SigmaPlot software, and results were expressed as means ± standard deviation (SD) or standard error (SE). To compare different groups of treatment, we use the non parametric Mann-Whitney runk sum test for *in vitro* studies and the Kruskal-Wallis H test (One way ANOVA on ranks) followed by Dunn's post hoc test for *in vivo* studies. Difference was considered significant with a *p* value ≤ 0.05.

## SUPPLEMENTARY MATERIALS FIGURES AND TABLES



## Abbreviations

MPM: Malignant Pleural Mesothelioma
MDM2: Murine Double Minute 2
rhTRAIL: recombinant human Tumor necrosis factor (TNF)-related apoptosis-inducing ligand
OS: Overall Survival
PFS: Progression Free Survival
MDMX: Murine Double Minute X
LOH: loss of heterozygosity
PI: Propidium Iodide
CI: combination index
PI3K: phosphatidylinositol3-kinase
mTOR: mammalian target of Rapamycin
HDAC: histone deacetylases
NFkB: Nuclear Factor kB
