# Supplementary material for: Synergistic targeting of malignant pleural mesothelioma cells by MDM2 inhibitors and TRAIL agonists
Source: Oncotarget. 2017 May 11;8(27):44232–41. doi: 10.18632/oncotarget.17790 (PMC5546476; doi:10.18632/oncotarget.17790)
Supplement: Supplementary file 1 [file oncotarget-08-44232-s001.pdf]

Synergistic targeting of malignant pleural mesothelioma cells by MDM2 inhibitors and TRAIL agonists

Supplementary Materials

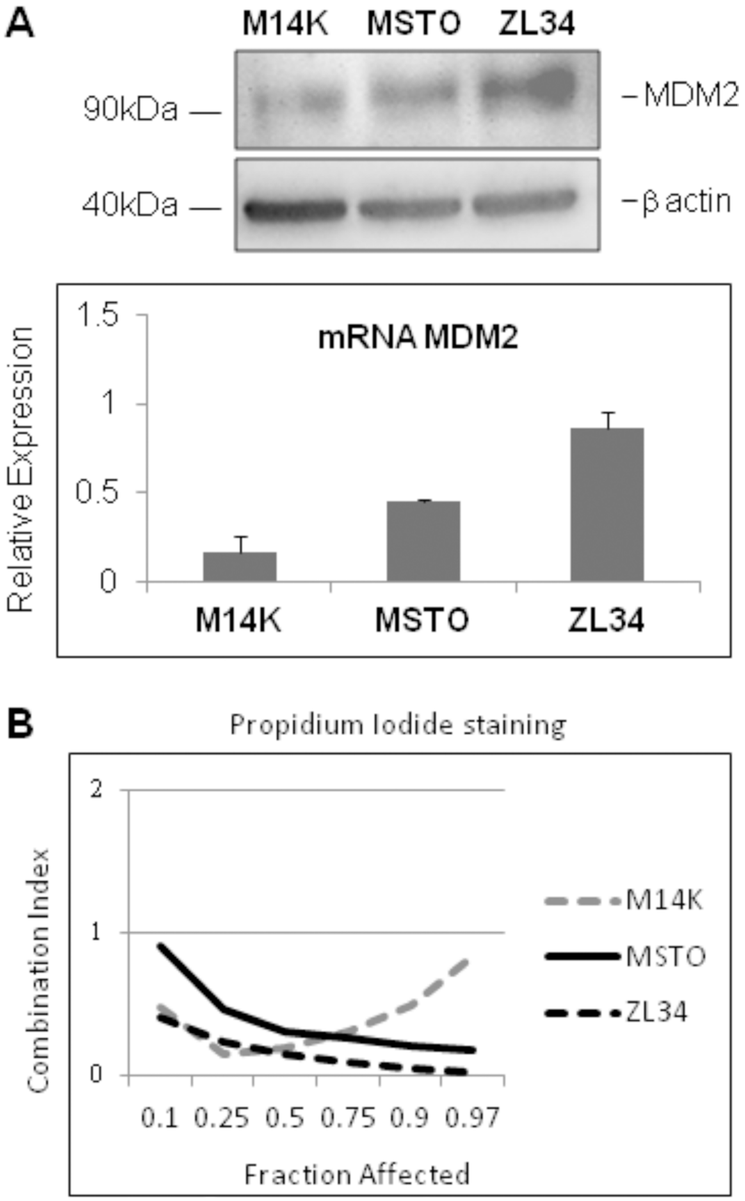

Supplementary Figure 1: Nutlin 3a plus rhTRAIL treatment in MPM cell lines. (**A**) Western blot (upper panel) and RT-PCR (lower panel) analysis of MDM2 protein and mRNA expression levels in untreated MPM cell lines. Western blot image is representative of two independent experiments; RT-PCR graph represent mean ± SE of relative mRNA expression of three different experiments running in duplicate. (**B**) MPM cell lines were treated for 24 hours with different concentration of Nutlin 3a and rhTRAIL (in constant ratio, 3.9:1). The synergism in apoptosis induction of the combinations was calculated using the Chou-Talalay Combination Index (CI) method.

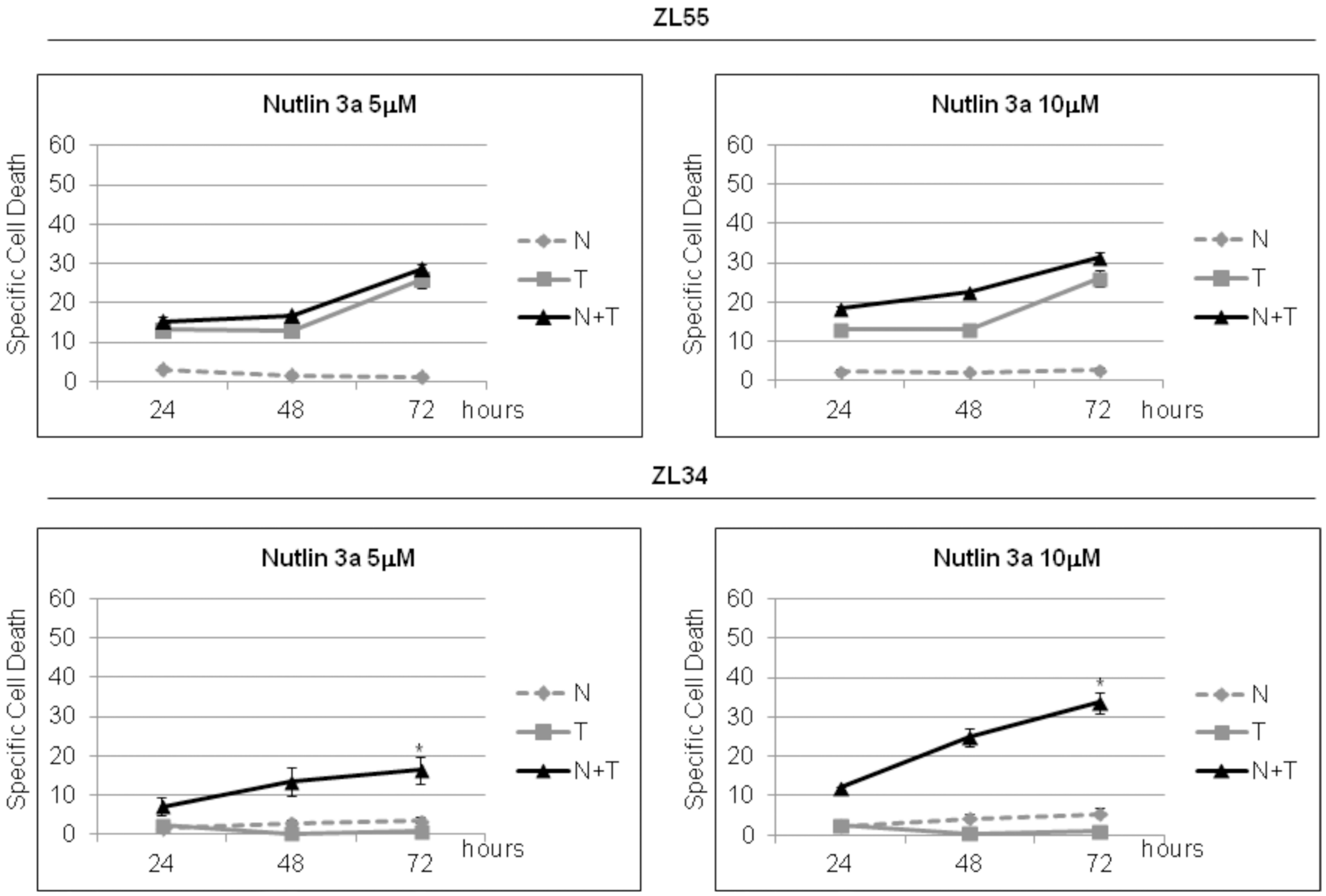

Supplementary Figure 2: Dose and time dependent apoptosis induced by Nutlin 3a plus rhTRAIL in MPM cells. ZL55 (upper panels) and ZL34 (lower panels) MPM cell lines were treated with DMSO or Nutlin 3a (5 or 10 µM) and/or rhTRAIL 2.56 µM for 24, 48 and 72 hours and PI Assay was performed. Results were represented as mean ± SE of SCD of two different experiments running in triplicate. Asterisks indicate statistically significant difference between rhTRAIL and Nutlin 3a *plus* rhTRAIL-treated cells ($p = 0.008$).

Supplementary Table 1: MPM STR profile

MARKERS	ZL55	M14K	MSTO211-H	ZL34
AMEL	X	X	XY	X
D3S1358	14	16	15	14–15
D1S1656	17.3	15–18.3	16.3–17	11–15
D2S441	10–11	14	10–14	11
D10S1248	13–14	13–14	14–16	14
D13S317	8–9	11	11–14	11–12
Penta E	12–18	11–16	7–13	5
D16S539	12	12–14	13	11
D18S51	13	17–20	16–18	14
D2S1338	19–21	25	20–24	23
CSF1PO	9–11	10–12	11–12	11–13
Penta D	10–14	11	11–12	13
TH01	6	6–8	8–9.3	6
vWA	15–19	17	16–18	18–20
D21S11	27–29	31.2–34.2	28–31	30
D7S820	7–11	9–10	8–12	9
D5S818	10–13	12	12	11
TPOX	8	8–9	11	8
DYS391	-	-	10	-
D8S1179	14	13–15	13	11–13
D12S391	17–19	18–19	18–19	18–21
D19S433	15.2–16	12–13	13–14	13–15
FGA	22	21–22	21	22
D22S1045	16	15	14–15	16
